# Trust and distrust in contradictory information transmission

**DOI:** 10.1007/s41109-017-0029-0

**Published:** 2017-06-05

**Authors:** Giuseppe Primiero, Franco Raimondi, Michele Bottone, Jacopo Tagliabue

**Affiliations:** 1 Department of Computer Science, Middlesex University, the Borroughs, London, NW4 4BT UK; 2 AXON VIBE, 524 Broadway, New York, NY10012 USA

**Keywords:** Trust, Distrust, Contradictory information, Agent-based simulation

## Abstract

We analyse the problem of contradictory information distribution in networks of agents with positive and negative trust. The networks of interest are built by ranked agents with different epistemic attitudes. In this context, positive trust is a property of the communication between agents required when message passing is executed bottom-up in the hierarchy, or as a result of a sceptic agent checking information. These two situations are associated with a confirmation procedure that has an epistemic cost. Negative trust results from refusing verification, either of contradictory information or because of a lazy attitude. We offer first a natural deduction system called SecureND^sim^ to model these interactions and consider some meta-theoretical properties of its derivations. We then implement it in a NetLogo simulation to test experimentally its formal properties. Our analysis concerns in particular: conditions for consensus-reaching transmissions; epistemic costs induced by confirmation and rejection operations; the influence of ranking of the initially labelled nodes on consensus and costs; complexity results.

## Introduction

Trusted information is an essential feature of computational contexts where agents might have to rely on external sources to execute decisions effectively and securely. With increasingly large networks, trust becomes a method to acquire information that would otherwise be unavailable or that is effectively hard to produce. Similarly, trust applies in contexts where a hierarchical structure is in place, defined for example by privileges in an access control model: here trust can be either a property of top-down communications, where information is not required to be confirmed; or the result of confirmation procedures in bottom-up transfers.

Besides network size and structure, another important factor that influences the result of trusted information sharing processes is the attitude of the network’s nodes, when these are understood as epistemic agents. Sceptic agents can be characterised by a requirement to check information; lazy ones by an attitude to reject it. This is of even greater relevance where contradictory information is allowed, e.g. in the context of opinion diffusion, belief propagation and its extreme cases, like fake news.

Hence, understanding conditions of information propagation and the costs related to topological and epistemic factors is crucial for dynamic (social) network analysis and access control models, with applications in mathematics, computer science, economics and biology, but also in concrete, less formal scenarios, e.g. (van de Bunt et al. 2005).

An efficient model of trust propagation highly depends, therefore, on the epistemic and structural features of the network in which trust is defined. Algorithmic models for efficient knowledge distribution based on trust relations are being investigated both formally and through applications. Despite the increasing literature, two important aspects have received little attention so far:

1. in contexts with contradictory information like social networks or insecure access control systems, understanding how positive and negative trust help or hinder the data flow;
2. the epistemic costs of (negative) trust transitivity.

In this paper we offer both a formal model and a computational simulation combining the above mentioned properties. The networks of interest are built by ranked agents, as they could occur in standard access control models. They are, moreover, defined in terms of an epistemic attitude:

- *sceptic agents* pay an epistemic cost for performing a checking operation before trusting the received information;
- *lazy agents* accept without checking information consistent with their current knowledge, while they distrust inconsistent messages.

In this context, positive trust is a property of the communication between agents required when message passing is executed bottom-up in the hierarchy, or as a result of a sceptic agent checking information. Negative trust is instead the result of rejecting received contradictory information. These two situations are associated with epistemic costs, essential to determine if a network that resolves contradictory transmissions by rejecting information is more or less costly than one which facilitates message passing by straightforward acceptance. We focus in particular on networks that preserve memory of previously obtained trusted communications.

First, we model the problem formally through a natural deduction system called SecureND^sim^, presenting rules that offer a proof-theoretical semantics for the behaviour of the agents. We formulate some meta-theoretical properties of its derivations, concerning in particular:

1. the computation of the trust value in a given derivation;
2. the resolution of derivations with contradictory information;
3. the convergence between valid formulae, order on agents and the application of specific rules.

The logic is an extension of the calculus for trust developed in (Primiero and Raimondi 2014) and extended in (Primiero 2016) with the semantics for negative trust from (Primiero and Kosolosky 2016). The calculus has been applied to problems in software management in (Boender et al. 2015a).

Second, we offer a NetLogo simulation that implements the algorithms underlying the calculus, maps formal derivations in various graphs and allows to test experimentally the formal properties. In our analysis we consider in particular:

1. changes in the final distribution of contradictory information in view of network topology;
2. changes in the final distribution of contradictory information in view of ranking and epistemic role of seeding agents;
3. the quantification of the epistemic costs for trust and distrust operations.

The experimental analysis offered here extends the initial results presented in (Primiero et al. 2017).

The paper is organised as follows. In “Related work” section we overview the related work, both formal and experimental. In “The logic (un)SecureND^*sim*^” section we introduce the calculus SecureND^sim^ and provide the meta-theoretical results. In “Design and implementation” section we introduce the principles underlying the graph construction and analyse the algorithms at the basis of the simulation. In “Experimental results” section we describe our experimental results on consensus, rankings, costs and complexity. Finally, “Conclusions” section presents general observations on our analysis and future work.

## Related work

Our work is at the confluence of several different research areas. We focus in particular on the role of trust in computational environments and the several approaches to its propagation. In relation to contradictory information, we overview the distinction between controversial users vs. controversial trust values. In qualifying our own semantic notion of trust, we report on models using binary and continuous trust values. Finally, we consider how local vs. global trust methods differ.

The first research area of interest concerns the treatment of computational trust for models of access control and network analysis. This area includes several logical model and algorithmic treatments, with an eye on applications and it spans several disciplines, including logics, cryptography, network theory, security protocols. Since the Bell-LaPadula Model in access control theory (Bell and LaPadula 1973), trust is intended as a property of agents, as opposed to security as a property of the system: trusted subjects are allowed to violate security constraints and trustworthiness of resources corresponds to prevention of unauthorised change. In more recent resource-based access control models, trustworthiness is either defined by temporal-spatial constraints (Chandran and Joshi 2005), or by user-defined constraints (Chakraborty and Ray 2006; Oleshchuk 2012). In authentication logics, trust is coupled to beliefs with application to distributed settings (Barker and Genovese 2011).

Trust propagation, interference and distrust blocking in uncertain environments and autonomous systems are receiving increasing attention (Carbone et al. 2003; Guha et al. 2004; Ziegler and Lausen 2005; Marsh and Dibben 2005; Jøsang and Pope 2005), with applications to internet-based services (Grandison and Sloman 2000), component-based (Yan and Prehofer 2011) and software management systems for security and reputation (Bugiel et al. 2011), or accuracy (Ali et al. 2013). A costs-efficient analysis in terms of modal logic is to be found in (Anderson et al. 2013). In the context of propagation, transitivity is a natural property to study. The problem of trust transitivity has received much attention, see (Christianson and Harbison 1997; Jøsang and Pope 2005) for an older and a more recent approach. An analysis for trusted communications in terms of dependent types is given in (Primiero and Taddeo 2012). In (Primiero and Raimondi 2014), trust is defined proof-theoretically as a function on resources rather than a relation between agents: this allows transitivity of writing privileges only under satisfaction of a consistency constraint. The calculus introduced in “The logic (un)SecureND^*sim*^” section is an extension thereof. Unfortunately, none of these previous logic-based approaches consider either the case of contradictory information, nor the aspects of epistemic costs associated with such a scenario. This task is the aim of the translation of logical principles in an experimental analysis, presented in “Design and implementation” section. Transitivity of trust is also analysed in the context of cryptographic applications, see e.g. (Maurer and Schmid 1996).

A related area of analysis is that of belief diffusion, especially in social networks. Continuous models assume a continuous numerical value on agents’ opinions, with updates depending on weights (Lehrer and Wagner 1981), where the latter can also vary over time (Chatterjee and Seneta 1977) or where influence is admitted only below a certain distance (Hegselmann and Krause 2002). A model that combines opinion diffusion with influencing power is presented in (Grandi et al. 2015). Another aspect of information diffusion in social networks which is gaining much attention is related to the propagation of misinformation or ‘fake news’, the resulting polarisation of communities and the possibility of cascades, see e.g. (del Vicario et al. 2016). Our model combines the comparative ranking value of agents with both their distinct epistemic attitudes and a majority selection in the case of conflicting information, which we take to indicate the presence of both a correct and an incorrect interpretation of facts.

In (Massa and Avesani 2005) controversial users are those generating a disagreement on their trustworthiness, either as the minimum between trust and distrust evaluations by other users, or as the difference in the number of trust and distrust judgements. The work in (Zicari et al. 2016) considers, instead, controversial trust values between two nodes, determined either as the trust weight of their edge, or as a fixed negative value when no path exists, or as a continuous value *t*∈[0,1] when there is no direct edge. Similarly, in our logic trust is a function on formulas obtained by verification, encoded in the network model by a property of edges when a node is labelled. Differently from the above, our model uses discrete values but it combines the comparative ranking of agents with both their epistemic attitudes and a majority selection in the case of conflicting information. The approach in (Massa and Avesani 2005) also uses a binary classification for users, so do several models for belief diffusion in social networks, with binary opinions for agents, considering neighbours’ influence (Granovetter 1978; Kempe et al. 2005) or majority (Raghavan et al. 2007). Trust defined by global methods is a value attached to a user and appropriate for a reputation evaluation at network level; in local methods, trust is inferred instead as a value between source and sink nodes, i.e., it is a feature of an edge. As it appears clearly from the above, our approach uses a local trust method in the case of non-conflicting information, resorting to a computation of trust using path lengths to determine which elements need to be distrusted in the case of conflicting information. This combination of features recalls the two controversial cases discussed in (Zicari et al. 2016): the *ToTrustOrNotToTrust* case resembles our binary choice, but moderated by continuous trust values, while we rely on ranking and epistemic attitudes; the *Asymmetric Controversy* case resorts to path lengths with preference for shortest paths, while we base our result on the number of distrustful edges present in each path.

The modelling and computer simulation of trust and its management for large (social) networks sharing data are also becoming widespread across various scientific communities. The exposure of users in social networks to ideologically diverse contents is a recent object of study in networks theories, see (Bakshy et al. 2015), in particular to qualify the assumption that such networks facilitate the creation of filter bubbles and echo chambers. Another example is given by the (standard and digital) scientific networks, which count trustworthiness as a parameter to select citations and co-authorship (Quattrociocchi et al. 2012). Accordingly, the role of trust in these types of networks is receiving much attention in academia, industry and policy-making. An overview of this research area with a focus on trust evolution is available from (Netrvalová 2006). Another extensive literature review of computational modelling of trust with a focus on evolutionary games and social networks is given in (Mui 2002). The role of trust in virtual societies is also analysed in (Coelho 2002) and (Corritore et al. 2003). A more recent analysis for social relations is provided in (Sutcliffe and Wang 2012). In (Iyer and Thuraisingham 2007) a Java simulation for trust negotiation and confidentiality between agents with common goals and only partial information is introduced. Transaction costs economics in inter-groups relations has been extended in view of trust in (Nooteboom et al. 2000): here agents attach a metric on trust relative to potential profit. Joint work in teams is also analysed in terms of trust to quantify performance at the individual and team level in (Martínez-Miranda and Pavón 2009).

As far as we are aware, none of the previous works combines a rule-based semantics with an experimental analysis. Moreover, the combination of characteristics of the model we analyse and simulate seem to have been ignored so far. These include: a characterisation of agents as sceptic or lazy towards epistemic contents; their structural ranking, typical of access control systems; the quantification of epistemic costs of trust and distrust; and the conditions for consensus in the presence of contradictory information.

## The logic (un)SecureND^*sim*^

In this section we introduce the logic (un)SecureND^*sim*^, a natural deduction calculus whose rules define how agents can execute access operations on (atomic) formulas and their negations. The proof-theoretic setting of the language has several advantages. First, it allows us to clearly express the algorithmic protocol introduced in “Design and implementation” section through pairs of rules that fully describe the semantics of each operation available to agents. Second, it provides the means to explore meta-theoretical properties of the model which the simulation cannot offer. Finally, it lays down preparatory work for the possible formal verification of the protocol.

Agents of the language are epistemically characterised and their different access privileges on contents are reflected in an order relation. We distinguish two types of agents who behave differently in the context of information transmission:

- *sceptic agents*: they pay an epistemic cost by performing a checking operation before trusting received information;
- *lazy agent*: they distrust the information when this is not consistent with their current knowledge.

Obviously, this distinction does not cover the whole spectrum of possible epistemic attitudes towards information received and it could be offered in a graded scale. We limit ourselves here to these two basic cases. While the logic is rigid with respect to how these attitudes are executed, in the simulation model developed in “Design and implementation” section, we allow for slight changes of behaviour, i.e. where we establish that only a certain fraction of sceptic agents check information.

Agents’ operations are those typical of access control models, namely reading and writing, where the former is here understood as message receiving, and the latter as message passing. They are further enhanced formally by the following operations:

- *verification*: it is required either by a top-down reading operation, i.e., when message passing is executed from below in the hierarchy; or by a reading operation performed by a sceptic agent;
- *falsification*: it is formulated as closure of verification under negation and it follows from reading contents that are inconsistent with the current knowledge of the receiver; or from a reading operation performed by a lazy agent;
- *trust*: is a function that follows from verification, when the content passed is consistent with the knowledge of the receiver;
- *distrust*: it is formulated as closure of trust under negation and it follows from falsification.

It is important to stress that, according to this operational semantics, agents do verify or falsify information on the basis of a contextual evaluation. Agents are here presented in a contextually empty process of information transmission and their evaluation is purely based on the evaluation of trustfulness and distrustfulness on the basis of criteria of majority and origin. In this sense, our logic, the algorithm and the simulation focus on the role of trust and distrust as independent from truthfulness criteria, while consistency requirements are crucial. In other words, we do not establish beforehand which of two contradictory atoms of information is true.

In line with a proof-theoretical approach, we define rules that allow to *introduce* a given function from premises, and one to *eliminate* it, i.e., to obtain a conclusion without such function. Such a pair of rules defines syntactically the meaning of access operations, including verification, trust and their negations. (un)SecureND^*sim*^ is here introduced as a stand-alone formal system, but it can be seen as a para-complete fragment of the logic SecureND presented in (Primiero and Raimondi 2014). This logic has been specifically designed to resolve situations of unintended (and therefore possibly risky) trust transitivity of the form:

*Alice trusts Bob and Bob trusts Carol; therefore Alice trusts Carol*.

In (Boender et al. 2015b), the logic is formally verified through translation to a Coq protocol and applied to a problem of trust transitivity in software management. In (Primiero 2016), the calculus is further extended with negation to define the logic (un)SecureND, which includes two negative trust protocols: one for misplacement of trust (mistrust), on for betrayal (distrust). (un)SecureND^*sim*^ models the latter in the context of message passing in a network of ordered agents with contradictory information. The extension to a verification protocol is a new property added to this family of logics by the present fragment.

We first introduce the syntax of the system and the main properties of the underlying access control model. We further illustrate in some details the rules and some minimal meta-theoretical properties of the related derivability relation. Finally we illustrate some structural properties of (un)SecureND^*sim*^ derivations concerning especially validity and the role of trust instances in the length of such derivations. These formal properties are later experimentally tested in “Experimental results” section.

### Formal preliminaries

#### **Definition 1**

The syntax of (un)SecureND^*sim*^ is defined by the following alphabet:

$$\begin{array}{l} V^{<}:= \{\mathtt{lazy(v_{i})}, \mathtt{sceptic(v_{i})}\}\\ \phi^{V}:= p^{v_{i}} \mid \neg \phi^{V} \mid Read\left(\phi^{V}\right)\mid Verify\left(\phi^{V}\right) \mid Write\left(\phi^{V}\right)\mid Trust\left(\phi^{V}\right)\\ \Gamma^{V}:=\{\phi^{v_{i}}_{1}, \dots, \phi^{v_{i}}_{n}\}; \end{array} $$

*V* ^<^ indicates the set of lazy and sceptic agents, each denoted by $\phantom {\dot {i}\!}v_{i},{v{j}},\dots $. The apex works as a formal reminder that agents are ordered according to an order relation <. The order relation < over *V*×*V* models the dominance relation between agents: *v*_*i*_<*v*_*j*_ means that agent *v*_*i*_ has higher relevance (e.g., in terms of security privileges) than agent *v*_*j*_. *ϕ* ^*V*^ is a meta-variable for boolean atomic formulae closed under negation and functions for reading, writing, verification and trust. It should be stressed that the current formal model and the subsequent algorithmic model refer to atomic information for simplicity, but the complexity of the formula representing the transmitted information is entirely irrelevant for our results. We use $\phantom {\dot {i}\!}\Gamma ^{v_{i}}$ to express a set of formulae typed by one agent *v*_*i*_∈*V*, typically the sender, in which a given formula *ϕ* ^*V*^ is derivable. $\phantom {\dot {i}\!}\Gamma ^{v_{i}}$ is called the *context* in which $\phantom {\dot {i}\!}\phi ^{v_{i}}$ is derived. We denote an empty context by ·⊩.

#### **Definition 2**

*(Judgement)* A judgement $\Gamma ^{v_{i}} \vdash \phi ^{v_{j}}$ states that a formula *ϕ* is valid for agent *v*_*j*_ in the context *Γ* of formulas (including operations) of agent *v*_*i*_.

Our judgements express thus some operation that the agent on the left-hand side of the derivability sign performs on information typed the agent on the right-hand side of the same sign. When message passing includes more than one agent, this is encoded in the system by an extension of the context, denoted as $\phantom {\dot {i}\!}\Gamma ^{v_{i}};\Gamma ^{v_{j}}$. A judgement stating the validity of a formula for one agent under a (possibly extended) context of formulas of (an)other agent(s) matches the procedure Transmission introduced below in “Design and implementation” section to extend a given graph *G* with a newly labelled vertex.

Assuming *v*_*i*_<*v*_*j*_, valid privilege transfers for access control in our system are summarised as follows by judgements of (un)SecureND^*sim*^:

- $\Gamma ^{v_{j}}\vdash Read(\phi ^{v_{i}})$: reading is always allowed when messages come from up in the order relation; this is not always the case in access control models, where one might establish reading privileges to hold only upwards, e.g. in the case where strict security is applied; we model a less strict scenario, where agents can always read information that is passed top-down.

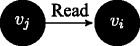

- If $\Gamma ^{v_{i}}\vdash Read(\phi ^{v_{j}})$ then $\Gamma ^{v_{i}}\vdash Verify(\phi ^{v_{j}})$: messages coming upwards from below in the order relation are passed on under a verification function.

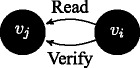

- If sceptic( *v*_*j*_ ), and $\Gamma ^{v_{j}}\vdash Read(\phi ^{v_{i}})$, then $\Gamma ^{v_{j}}\vdash Verify(\phi ^{v_{i}})$: we further wish to enhance the structure of the model by requiring that the message passing is qualified by verification in one additional case: not only because the transmission is executed upwards in the dominance relation, but also because a sceptic agent is on the receiving side and it verifies the information.

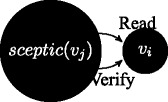

- If lazy( *v*_*j*_ ), and $\Gamma ^{v_{j}}\vdash Read(\phi ^{v_{i}})$, then $\Gamma ^{v_{j}}\vdash \neg Verify(\phi ^{v_{i}})$: when a lazy agent is on the receiving side, information is not verified.

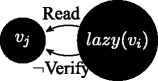

- If $\Gamma ^{v_{i}}\vdash Read(\phi ^{v_{j}})$ and $\Gamma ^{v_{i}}\vdash \neg \phi $, then $\Gamma ^{v_{i}}\vdash \neg Verify(\phi ^{v_{j}})$: when a content read from below contradicts current knowledge, refutation is modelled as negation of verification.

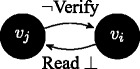

**Fig. 1:**
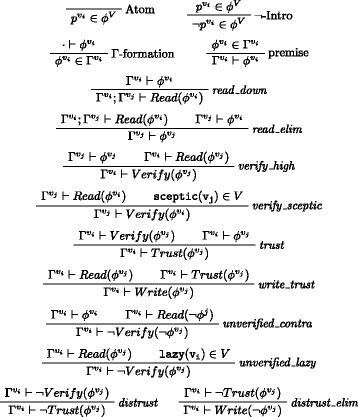
The system (un)SecureND^*sim*^

Notice that in the last two cases refuted verification leads to negated trust. The rule system (un)SecureND^*sim*^ modelling these cases is introduced in Fig. 1 and it assumes that *v*_*i*_<*v*_*j*_ holds. The first rules are for inductive construction of a context $\phantom {\dot {i}\!}\Gamma ^{v_{i}}$. Any such context, also called user profile, is required to be consistent (i.e., it admits only one of *ϕ* and ¬*ϕ*) after a message passing operation is concluded. We use a standard context-extension syntax $\phantom {\dot {i}\!}\Gamma ^{v_{i}}; \phi ^{v_{j}}$ to indicate that the extension of the profile for agent *v*_*i*_ with a formula *ϕ* from agent *v*_*j*_ preserves consistency. Extensions that do not preserve consistency are not allowed. The rule *read*_*down* establishes that if a message is owned by a user *v*_*i*_ it can be read downwards (this is the first of the above privilege transfers). The rule *read*_*elim* is the corresponding elimination rule: a message that is read (first premise) and preserves consistency (second premise) can be owned, expressed by the change of label in the formula *ϕ*. We now consider the case of access from upwards in the dominance relation. The rule *verify*_*high* says that if a message owned by an agent *v*_*j*_ is read from another agent *v*_*i*_ higher in the dominance relation, then a verification action is required. Similarly, by rule *verify*_*sceptic* a verification procedure is called when the receiver is sceptic (independently from the order, hence also when the receiver is below). The rule *trust* is the elimination rule for the verification procedure: if *verify* is called and the message preserves consistency (i.e., is derivable in the current agent’s profile), then the link between the agents about that message is trusted. Trust elimination corresponds to message passing according to *write*_*trust*. The current simple setting of (un)SecureND^*sim*^ models information rejection through a consistency checking rule: every message passing operation is eventually accepted if consistent. Verification, and accordingly Trust, are not implemented in two cases: by rule *unverified*_*contra* when messages received conflict with currently held contents; and by rule *unverified*_*lazy* when when a lazy agent is on the receiving side. Resolution of two received contents inconsistent with one another are resolved below through an additional definition based on popularity (see Definition 5). Missing verification implies *distrust*, and this in turn passing the opposite message by *distrust*_*elim*.

#### **Example 1**

A simple derivation of message passing (assuming *v*_*i*_<*v*_*j*_):


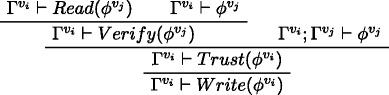


This derivation illustrates a message *ϕ* written by agent *v*_*j*_, read by agent *v*_*i*_, verified and written to be passed further on. This standard case holds also if *v*_*i*_∈sceptic_node (even assuming *v*_*j*_<*v*_*i*_); if *v*_*i*_∈lazy_node, then the verification passage is skipped to infer *write* directly.

#### **Proposition 1**

Any successful (un)SecureND^*sim*^ message-passing operation is a derivation tree including a Write-Read-(Verify-Trust)-Write series of sequents.

Standard logical notions can be formulated as follows:

#### **Definition 3**

*(Satisfiability)* An (un)SecureND^*sim*^ judgement $\Gamma ^{v_{i}}\vdash \phi ^{v_{i}}$ is satisfied if there is a derivation *D* and a branch *D* ^′^⊆*D* with a final step terminating with such a judgement.

#### **Definition 4**

*(Validity)* An (un)SecureND^*sim*^ judgement *Γ* ^*V*^⊩*ϕ* ^*V*^ is valid if there is a derivation *D* and for all branches *D* ^′^⊆*D* and for all agents *v*_*i*_∈*V*, there is a final step terminating with such a judgement.

### Structural properties on derivations

By Proposition 1, verification and trust are optional steps in a derivation if the message is received by a lazy agent. This suggests that each derivation (or branch thereof) can be analysed in view of its length to count the number of trust rule instances occurring in it.^1^ This allows us to identify the number of times an atomic message *ϕ* has been trusted in a given derivation *D*. We denote such measure by $\phantom {\dot {i}\!}\mid \!{Trust}(\phi ^{V})\!\mid _{D}$.

#### **Theorem 1**

∣ *Trust*(*ϕ* ^*V*^) ∣_*D*_=∣ *Verify*(*ϕ* ^*V*^) ∣_*D*_, for all *v*_*i*_∈*V*.

#### *Proof*

By induction on the length of *D*, provided that *verify*_*high* and *verify*_*sceptic* are the only rules that introduce a formula $\phantom {\dot {i}\!}Verify(\phi ^{v_{i}})$ which is the premise of a *trust* rule. □

This computable method allows us to offer a simple resolution for the case in which consistency fails and one wants to decide on the basis of the more trusted formula on the derivation tree.

#### **Definition 5**

*(Conflict Resolution by Trust Majority)* Given a derivation *D*_1_ terminating in $\Gamma ^{v_{i}}\vdash Write(\phi ^{v_{i}})$ and a derivation *D*_2_ terminating in $\Gamma ^{v_{j}}\vdash Write(\neg \phi ^{v_{j}})$, a new step holds which takes as premises $\Gamma ^{k}\vdash Read(\phi ^{v_{i}})$ and $\Gamma ^{k}\vdash Read(\neg \phi ^{v_{j}})$ respectively, and concludes $\Gamma ^{v_{k}} \vdash \phi ^{v_{k}}$ if and only if $\mid \!Trust(\phi ^{V})\!\mid _{D_{1}} > \mid \!Trust(\neg \phi ^{V})\!\mid _{D_{2}}$.

This suggests that at any stage of branch merging, the most popular (trusted) content is preserved, hence enforcing a network effect.

A different resolution strategy can be enforced by computing the number of times an atomic message *ϕ* has been distrusted in a given derivation *D*. We denote such measure by ∣ *DisTrust*(*ϕ* ^*V*^) ∣_*D*_.

#### **Theorem 2**

∣ *DisTrust*(*ϕ* ^*V*^) ∣_*D*_=∣ ¬*Verify*(*ϕ* ^*V*^) ∣_*D*_, for all *v*_*i*_∈*V*.

#### *Proof*

By induction on the length of *D*, provided that *unverified*_*contra* and *unverified*_*lazy* are the only rules that introduce a formula $\phantom {\dot {i}\!}\neg Verify(\phi ^{v_{i}})$ which is the premise of a *distrust* rule. □

#### **Definition 6**

*(Conflict Resolution by Distrust Majority)* Given a derivation *D*_1_ terminating in $\Gamma ^{v_{i}}\vdash Write(\phi ^{v_{i}})$ and a derivation *D*_2_ terminating in $\Gamma ^{v_{j}}\vdash Write(\neg \phi ^{v_{j}})$, a new step holds which takes as premises $\Gamma ^{k}\vdash Read(\phi ^{v_{i}})$ and $\Gamma ^{k}\vdash Read(\neg \phi ^{v_{j}})$ respectively, and concludes $\Gamma ^{v_{k}} \vdash \phi ^{v_{k}}$ if and only if $\mid \! DisTrust(\phi ^{V})\!\mid _{D_{1}} < \mid \! DisTrust(\neg \phi ^{V})\!\mid _{D_{2}}$.

From Definition 4, the following holds:

#### **Lemma 1**

For each (un)SecureND^*sim*^ derivation *D* with a valid formula *Γ* ^*V*^⊩*ϕ* ^*V*^, there is a graph *G* that is unanimously labelled by *ϕ*.

#### *Proof*

The proof requires to construct a graph *G* with a node for each distinct *v*_*i*_∈*V* occurring in *D* and an edge for each judgement instantiating one or more rules with two distinct nodes on each side of the derivability sign. Starting from the node occurring at the highest position of *D* validating *ϕ*, by application of one or more sequences of rules the conclusion in such branch of *D* represents a new node in *G* labelled by *ϕ*. If all branches of *D* terminate with a formula validating *ϕ*, as by assumption and according to Definition 4, then all nodes in *G* will be labelled by *ϕ*. □

The construction of such a graph *G* for experimental purposes is the aim of “Related work” section. Notice, moreover, the following structural properties on SecureND ^*sim*^ derivations.

#### **Lemma 2**

For a derivation *D* of (un)SecureND^*sim*^, the value of ∣ *Trust*(*ϕ* ^*V*^) ∣_*D*_ is directly proportional to the number of *verify*_*high* rule applications and the number of distinct sceptic( *v*_*i*_ )∈*V* occurring as labels in the premises of the derivation.

#### *Proof*

By structural induction on *D*, selecting the appropriate step as indicated by Theorem 1. □

#### **Lemma 3**

For a derivation *D* of (un)SecureND^*sim*^, the value of ∣ ¬*Trust*(*ϕ* ^*V*^) ∣_*D*_ is directly proportional to the number of *unverified*_*contra* rule applications and the number of distinct lazy( *v*_*i*_ )∈*V* occurring as labels in the premises of the derivation.

#### *Proof*

By structural induction on *D*, selecting the appropriate step as indicated by Theorem 2. □

#### **Lemma 4**

Given a (un)SecureND^*sim*^ derivation *D*, the formula $\Gamma ^{v_{i}}\vdash \phi ^{v_{i}}$ converges to validity in *D* and to full labelling in the corresponding graph *G* as a direct function of:

- the number of instances of the *verify*_*high* rule applications.
- the number of instances of the *verify*_*sceptic* rule applications, for each *v*_*i*_∈*V*.

where $\phantom {\dot {i}\!}\phi ^{v_{i}}$ occurs in the conclusion, and as an inverse function of:

- the number of instances of the *unverified*_*contra* rule applications.
- the number of instances of the *unverified*_*lazy* rule applications, for each *v*_*i*_∈*V*.

where $\phantom {\dot {i}\!}\phi ^{v_{i}}$ occurs in the first premise.

#### *Proof*

This follows directly by Lemmas 2 and 3, and for the graph analysis by Lemma 1; the more verification operations and the more sceptic agents, the higher the convergence towards validity; the more distrust operations on the same formula, and the more the lazy agents, the lower the convergence. □

We offer in the following sections an agent based simulation which implements the set of rules described in (un)SecureND^*sim*^ and proceed with an experimental analysis of its conditions and results.

## Design and implementation

In this section we illustrate the design and implementation of a NetLogo model (Wilensky 1999) based on (un)SecureND^*sim*^ to investigate properties related to knowledge distribution depending on the epistemic attitude of the seeding agents and on the network topology. NetLogo is a well-known, widely used modelling platform for complex systems of interacting agents.

We start with basic definitions and analysis of the topologies of interest.

### **Definition 7**

*(Graph)* A network is an undirected graph *G*=(*V,E*), with a set *V*={*v*_*i*_,…,*v*_*n*_} of vertices representing our agents and a set *E*={*e*_(*i,j*)_,…,*e*_(*n,m*)_} of edges, representing transmissions among them.

### **Definition 8**

*(Labelling)* Each vertex *v*_*i*_∈*V* can be labelled by formulas as follows:

- *v*_*i*_(*p*) denotes a vertex labelled by an atomic formulas and expresses an agent *i* knowing *p*;
- *v*_*j*_(¬*p*) denotes a vertex labelled by the negation of an atomic formula and expresses agent *j* knowing ¬*p*;
- *v*_*k*_() denoted a vertex with no label and expresses an agent *k* who does not hold any knowledge yet.

An edge between two labelled nodes is denoted by *e*(*v*_*i*_(*p*),*v*_*j*_()) and denotes a transmission channel from agent *i* to agent *j* such that the former can transmit *p* over to the latter. The case *e*(*v*_*i*_(*p*),*v*_*j*_(¬*p*)), i.e., where an edge is constructed between two agents holding contradictory information, is admissible and it requires a resolution procedure. This is generalised below to the case where one agent who does not hold any knowledge yet receives contradictory information from two distinct agents. To this aim, a non-standard notation with three nodes *e*(*v*_*i*_(*p*),*v*_*j*_(),*v*_*k*_(¬*p*)) is used in the following to abbreviate the presence of two edges *e*(*v*_*i*_(*p*),*v*_*j*_()) and *e*(*v*_*k*_(¬*p*),*v*_*j*_()) between three nodes, one holding *p*, one holding ¬*p* and one with no information. This is another case where a node with an empty label requires a resolution procedure to choose between labels *p* and ¬*p*.

The order relation ≤ over *V*×*V* from the logic (un)SecureND^*sim*^ is preserved in our implementation. Such order becomes total or partial in view of the different possible topologies of the network: total, linear, random, or scale-free.

**Fig. 2:**
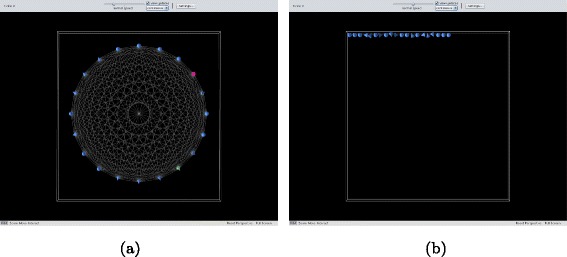
**a** A total network. **b** A linear network
- In a linear network, each vertex has an edge to the next vertex higher in the ranking; by transitivity, this order is also total. See Fig. 2 b.
- In a random network, for as long as new nodes are introduced, edges are created making sure that for each vertex at least one edge with another vertex is established; the ranking is here assigned by nodes labelled at the beginning (the seeding node) and never overwritten, the order is partial. See Fig. 3 a.

- In a total network, each vertex has an edge connecting it to any other vertex and equal ranking is assigned to all agents; the underlying dominance relation is then a total order. See Fig. 2 a.

**Fig. 3:**
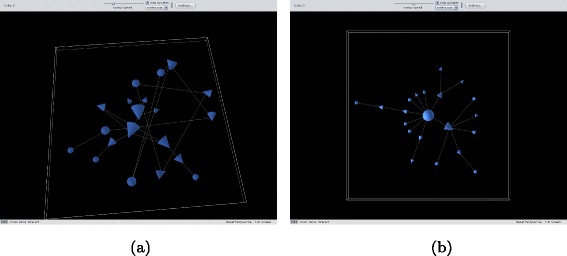
**a** A random newtork. **b** A scale-free network
- Scale-free networks (Milgram 1967; Watts and Strogatz 1998) use the Barabasi-Albert method to establish edges. Initialised by *m*=3 nodes, each node with 0 neighbours is asked to create an edge with a vertex in the network; for each new vertex *v*_*j*_ without neighbours, *v*_*j*_ is connected to up to *n*<*m* existing vertices with a probability **p**(*v*_*j*_) defined by the following expression:
  $$\mathbf{p}({v_{j}}) = \frac{k_{v_{j}}}{\sum_{v_{i}} k_{v_{i}}} $$
  where $k_{v_{j}}$ is the number of neighbours of agent *v*_*j*_ and the sum is made over all pre-existing nodes *v*_*i*_. Newly added nodes tend to prefer nodes that already have a higher number of links. The ranking in this case is given to each node by a simple function $\frac {1}{\mid edges\mid }$. Scale-free world networks are characterised by the clustering coefficient with a degree distribution that follows a power-law. See Fig. 3 b.

The maximum number of vertices in our graphs is set at 300. The scale-free network model can be assumed to be representative of real social networks cases: the assumption is that the degree distribution (as encoded in the network topology) is the main factor to be investigated by the model. It is known that many global network properties (like resilience to attack, short distance between any pair of nodes etc.) depend on network structure as defined much more than network size, so it is a reasonable starting point for our model. As most interesting real-world networks follow the scale-free model, the analysis of real world networks concerning trust and information spreading can be sufficiently modelled by this network type.

**Fig. 4:**
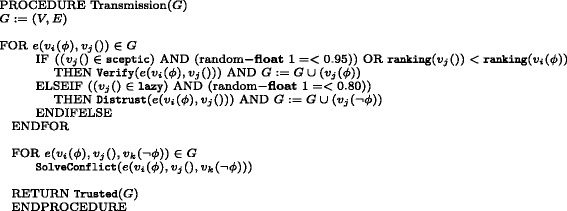
Algorithm for simple information transmission

The randomly seeded contradictory information *p*,¬*p* spreads across the network, according to the algorithm Transmission in Fig. 4: for each edge between a node labelled by *p* and one with an empty label, if the latter node is sceptic and in this case if it is part of a prefixed 95% of the sceptic population, or if its ranking is higher than that of the sender, then it calls the Verify routine and it is added to the network with the new label *p*; notice that here we have implemented a random selection of a 5% of sceptic agents who do not ask for verification; if the receiver is lazy and it is part of a prefixed 80% of the lazy population, it calls the Distrust routine and the new node is added with an opposite label; again, we have here set a random 20% of sceptic agents who do not distrust the information. Hence, the epistemic description of our agents follows the basic distinction between lazy and sceptic agents introduced in Section “The logic (un)SecureND^*sim*^”. But, as mentioned above, to offer a more realistic description of our agents and the network they form, we allow change of attitude. In particular, we allow a very low rate of verification cases for lazy agents in networks that have a majority of such agents; and a similar rate for sceptic agents that might accept information without implementing verification in networks that have a balanced distribution of lazy and sceptic agents. The rationale behind this choice is as follows: we assume that in a network with a large majority of agents with a virtuous behaviour, this is preserved; in a balanced network, we allow a low number of virtuous agents to slip in their habit; and in a network largely characterised by the lazy behaviour, we still allow some of the agents to be influenced by the few ones that have a sceptic attitude. To realise this design, we implement three fixed distributions of this epistemic attitude across the networks and a semi-random implementation of the corresponding procedures. Hence, we define three configurations of networks:

1. *overly lazy network*: in this type of network, the proportion of sceptic nodes is set at 20%, with their confirmation rate at 5%, the latter expressing the proportion of such agents that will after all ask for verification;
2. *balanced network*: in this type of network, the proportion of sceptic nodes is set at 50% and their confirmation rate at 95%, to account for a 5% of random sceptic agents who decide not to ask for verification after all;
3. *overly sceptic network*: in this last type of network, the proportion of sceptic nodes is set at 80%, their confirmation rate at 100%, hence verification is always implemented.

**Fig. 5:**
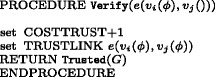
Algorithm for trust costs increase

**Fig. 6:**
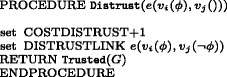
Algorithm for distrust costs increase

The sub-routine Verify is illustrated in Fig. 5: its role is to increase the value of costs associated with the number of *trusted links*. A successful confirmation procedure establishes trust as a property characterising edges and it equals to 0 at set up stage. Accepting information means in turn to create a trusted edge (marked green in the simulation) and to acquire knowledge of the atom passed. A graph *G* is relabelled to Trusted(*G*) by the procedure Transmission. Notice that passages where the receiver agent is lazy or lower in the dominance relation do not generate a *trusted link*.^2^ The subroutine Distrust is illustrated in Fig. 6: it increases the value of costs associated with the number of *distrusted links* and it labels the receiving node with the atom contrary to the one received.

When a node labelled by an atom *p* (by a previous interaction) is linked to another node labelled by the contradictory ¬*p*, the routine SolveConflict is started. We offer here two versions of this resolution strategy.

**Fig. 7:**
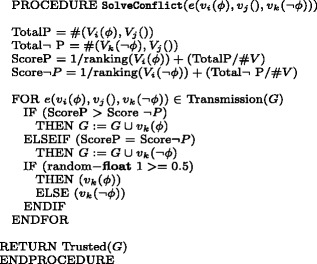
Algorithm for conflict resolution by trust majority
- The second version, see Fig. 8, analyses the number of distrusted links appended to each neighbour with each contradictory piece of information and it selects the new label from the least distrusted one, proceeding by random choice when an equal number of distrusted links is detected. We assume here a context in which agents refer to a popularity criterion in order to choose which of two contradictory pieces of information *not* to preserve. It then executes the subroutine Distrust on the selected link.

- The first one, see Fig. 7, takes into account the number of links with nodes labelled by *p*, the number of links with nodes labelled by ¬*p* and sums them to the respective overall rankings, obtaining values Score P and Score¬P. This implementation sensibly refines the pure majority by counting of the formal system in “The logic (un)SecureND^*sim*^” section, by adding the ranking of the agents involved as a parameter of the related score. For each pair of edges from nodes with contradictory information *p*,¬*p* to an unlabelled node, if the value of scoreP is higher than the value of score¬P, the new node is labelled by *p*, by ¬*p* otherwise. We assume here a context in which agents refer to a popularity criterion in order to choose which of two contradictory pieces of information to preserve.

**Fig. 8:**
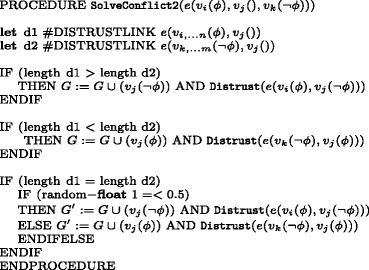
Algorithm for conflict resolution by distrust majority

The observer-property of *trustworthiness* (i.e., the total number of trusted links) and *distrustfulness* (i.e., the total number of distrusted links) are a known property of the network at any given time and used to perform conflict resolution. By the procedure clearP, trusted and distrusted links obtained by a first message passing operation are preserved in subsequent executions of the procedure Transmission over the same graph to analyse their effect on epistemic costs. An objective of the experimental analysis in “Experimental results” section is to compare results of the two resolution sub-routines to determine the effects of distinct conflict resolution strategies based on trust and distrust.

## Experimental results

Experiments are run over the four distinct types of networks. Scale-free networks better represent the topology of complex graphs as they occur, for example, in social networks. On the other hand, linear networks are more common in hierarchical structures that can be encountered in conditions of access control. The experiments have been executed on a machine with 7.7 GB of memory running 64bit Ubuntu 15.10. We have collected data from several scale-free networks of fixed dimensions between 10 and 300 nodes. The seeding of contradictory information is done by associating an atom *p* to a lazy node, and its negation to a sceptic one (although this association can be altered at will). The code and result of the experiments are available at https://github.com/gprimiero/securendsim.

A first set of experiments has been done on what we call a *memoryless network*. In this case, at each successive run of the algorithm the structure of the graph is re-plotted. As a result, all previous trusted edges are forgotten, meaning that the next information distribution is not affected by the previously established links. For this type of networks we have run a total of 240 executions of the main algorithm, 30 for each size of 10,20,30,40,50,100,200,300 nodes. The following results can be consistently observed:

- The knowledge plot, i.e., the final labelled graph is never consistent with the previous execution.
- There is no systematic distribution of consensus across the 30 runs.
- There is no systematic relation between the resulting knowledge plot and the costs of the transmission.
- There is no systematic relation between the knowledge plot and the ranking of the seeding nodes (nodes labelled at the beginning).

This indicates that memoryless networks do not offer a reliable experimental setting to investigate issues of consensus and epistemic costs of trusted graphs.

The second set of experiments has been performed again on several networks of different size, but ensuring at each run of the main algorithm that the trust graph obtained by a previous run is preserved. This is obtained by executing at the end of each execution a procedure clearP, which eliminates all labels from the graph, but preserves ranking and trusted links. In this way we can better average on the number of trusted and distrusted links which are created and destroyed at each execution.

Under these experimental conditions, we analyse consensus, costs, ranking of the seeding nodes and time complexity in networks with trust and distrust.

### Consensus

Here and in the following we will call a graph that satisfies consensus a *unanimous graph*.

**Fig. 9:**
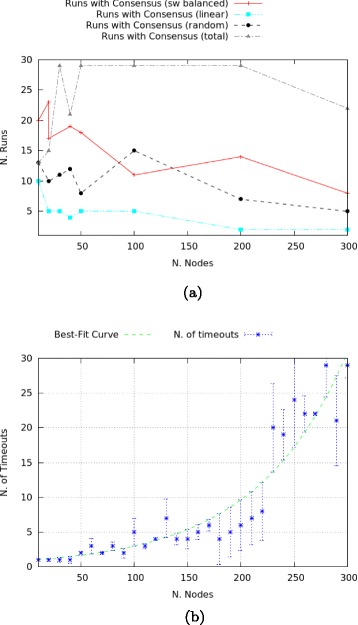
**a** Comparison of consensus between networks with trust. **b** Proportion of timeouts in random networks

Network configuration directly affects consensus results in memory-preserving networks with trust only, see Fig. 9 a, summarised as follows:

- Total networks reach consensus most often.
- Scale-free networks always perform better than linear or random ones in terms of number of runs that reach consensus.
- The data for random networks is not overly reliable, as full labelling might not be reached (increasingly often in the number of nodes). For this reason, a timeout is set at 1000 steps. A step indicates here one message passing operation. The proportion of runs that timeout is given in Fig. 9 b, showing a non-strictly linear increase. Accordingly, the number of runs that reach consensus is bound to decrease.

**Fig. 10:**
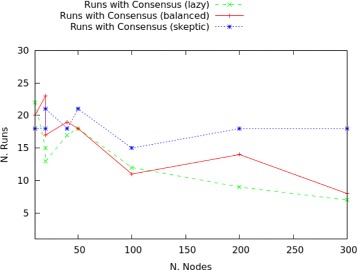
Consensus in scale-free networks

In particular, for scale-free networks the clustering of lazy nodes is inversely proportional to the construction of trusted edges and in larger networks disadvantageous to consensus reaching transmissions. Increasing the number of nodes while keeping the proportion sceptic-lazy constant, means also to increase the probability of clusters of lazy nodes: this means to reduce the number of trusted edges, in turn progressively reducing the number of runs in which consensus is obtained. Results for the three configurations (lazy, balanced and sceptic scale-free networks) are plotted in Fig. 10. These results show that in smaller networks (10 to 30 nodes), clusters of lazy nodes occur relatively often: the results for the three configurations are grouped in a restricted area, between 13 and 23 runs with consensus over 30. In these small networks, the denser groups of lazy nodes balance the reduced number of trusted links by sceptics. Once network size increases, the positioning of lazy nodes becomes more sparse. This topological factor crucially influences the number of runs in which consensus is reached: in general, overly sceptic networks perform better at becoming unanimous graphs. This can also be interpreted by saying that lazy nodes are less strict in preserving their labelling (i.e., they denote agents who are more prone to change their minds).

**Fig. 11:**
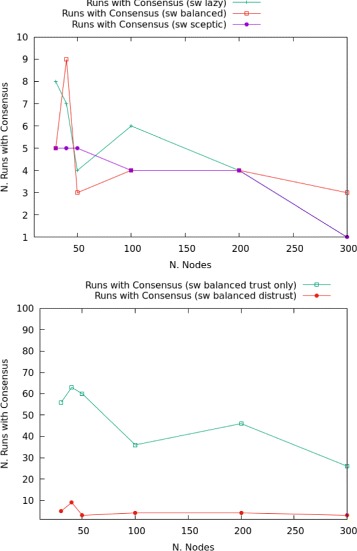
Consensus in scale-free networks with distrust

As shown in Fig. 11, networks with trust *and* distrust present an inverse correlation between size and the number of transmissions that reach consensus: the smaller the network, the more often full labelling with a unique formula is obtained (i.e., it is easier to reach consensus). Despite some differences in the reached peaks by lazy and balanced networks, the behaviour is overall similar in all configurations: balanced networks have the highest absolute number of such runs, while networks with higher proportion of sceptic agents have the lowest number of consensus reaching transmissions. Networks with distrust significantly differ from those with trust only for the total amount of consensus-reaching transmissions. We show this for balanced networks in the second graph of Fig. 11, the same holding true for lazy and sceptic networks: the presence of a distrust routine has a strong impact on the ability of the network to reach consensus in the presence of contradictory information, with no more than 9% of runs reaching a full labelling by either *p* or ¬*p* (network of 40 nodes), while in the case of networks with trust only this value reaches 63% (for networks of the same size).

These experimental results on consensus support empirically the properties of SecureND^sim^ derivations provided in “Structural properties on derivations” section, Lemma 4. To observe this, consider that total networks are graphs in which the number of edges between nodes is maximal, corresponding to derivations with the maximal number of branches, one for each pair of agents (*v*_*i*_,*v*_*j*_) appearing respectively in the premises and in the conclusion. Similarly, overly sceptic networks are graphs corresponding to derivations where more instances of the *verify*_*sceptic* rule are used. In both cases, the number of executions resulting in consensus are maximal, as stated by the first item in Lemma 4. On the other hand, linear networks are graphs corresponding to derivations where the number of agents for which the ranking can be transitively established is maximal, and overly lazy networks are graphs corresponding to derivations where more agents implement the *unverified*_*lazy* rule.

### Epistemic costs

The second type of experimental analyses concerns epistemic costs. With this term we refer to the computational expenses required to perform verification and distrust operations: these correspond in the calculus to instances of rules *verify_high* and *verify_sceptic* for trust and rules *unverified_contra* and *unverified_lazy* for distrust; in the algorithm they correspond to Verify and Distrust procedures. The effect of these procedures in the network is to generate trusted and distrusted links respectively. Given that there are proportionally more nodes than links and a message might pass more than once over a given node (through several senders), the values for costs are expected to be higher than those for links. Moreover, given the conditions for Verify are more than those for Distrust, the values of trust can be expected to be higher than those for distrust. The aim is to asses these values in the different topologies, to evaluate the proportion between trust and distrust costs and to use them as parameters to evaluate these actions with respect to consensus and complexity.

**Fig. 12:**
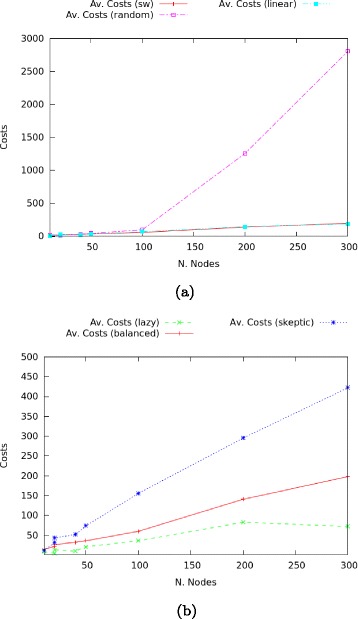
**a** Comparison of average costs of trust. **b** Average costs of trust in scale-free networks

We start by comparing balanced scale-free with random and linear networks with trust only, see Fig. 12 a. The results can be summarised as follows:

- Random networks are by far the most epistemically expensive.
- Linear networks are slightly less expensive than scale-free ones, by a very small margin. If one is balancing costs against information diffusion, scale-free should always be preferred to linear networks, as the associated costs do not diverge much (in general, scale-free networks are more resilient than linear ones).
- Given the previous observation on consensus and time-outs, it is obvious that random networks are the worse performing ones.

We now compare in more detail average trust costs in scale-free networks in all configurations (balanced, overly lazy and overly sceptic). The results are plotted in Fig. 12 b. By definition, a network with a higher number of sceptic nodes and confirmation requests will have higher trust costs. For small networks up to 40 nodes the costs are within a small range between 9 and 52; the difference increases significantly between larger overly lazy and overly sceptic networks. Cost difference remains comparably restricted for balanced and overly lazy networks (with a minimum difference of 15 average points at 50 nodes). This suggests that if one is trying to balance trust costs against consensus, large lazy networks should be preferred over balanced ones, as in the latter case the number of runs with uniform labelling tends to drop, while the costs still increase.

**Fig. 13:**
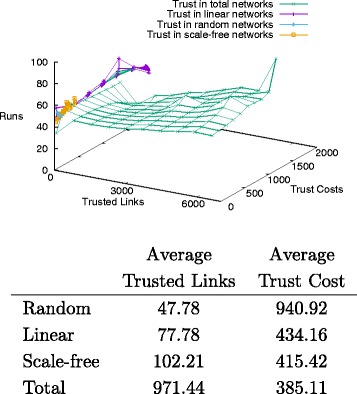
Trust distribution and average costs

Figure 13 and the associated Table show that the average rate of links and trust costs is inversely proportional: the former increases from random, through linear, scale-free and total networks, while the latter decreases. Given the fixed number of sceptic agents across the various topologies, the decrease in costs should be mainly associated with the ranking of agents and their order, while the increase in trusted links is purely due to the number of links in the network. From these data it appears that random networks perform the worst, as the required costs are high but the obtained links are less than in scale-free or linear networks.

**Fig. 14:**
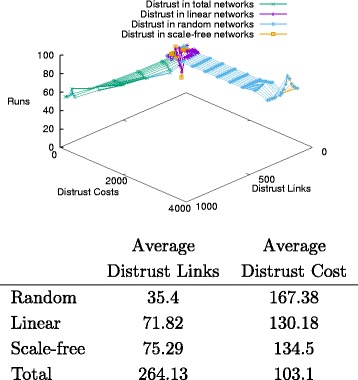
Distrust distribution and average costs

The different topologies show a similar pattern with respect to distrust values. As shown in Fig. 14 and the associated Table of average values, random networks are the most expensive with respect to distrust, and have the lowest number of distrusted links; linear networks remain constrained in the number of distrusted links, with costs decreasing; scale-free networks do not show a sensibly better behaviour, with comparable number of distrusted links and costs; finally, total networks perform the best, with the highest levels of links and relatively lower costs. As shown in the graph, it is remarkable the diverging behaviours of total and random networks: the former ones have almost stable distrust costs with increasing distrusted links, while the latter have stable links with increasing costs.

The comparison between tables shows that the average number of trusted and distrusted links grows in parallel, while the related costs decrease in a similar vein across the different topologies. Nonetheless, this proportion is not linear. Trust propagates at a much higher rate than distrust in these balanced networks, and there is a small difference between scale-free and linear networks, where the former presents more distrust than trust cost when compared to the latter. These observations suggest that trust is a more frequent and more relevant property in information transmission than the latter in general, and that linear networks are less affected than scale-free ones by distrust propagation.

Let us briefly compare these experimental results with theoretical properties of SecureND^sim^ derivations from “Structural properties on derivations” section. Lemma 2 states that, given a fixed number of sceptic agents in a derivation, the resulting value of trust instances, defined as epistemic costs, is only due to the applications of the *verify_high* rule. The applications of the rule in question map directly to the number of order relations satisfied by agents in the derivation, and hence to the number of agents that are higher in the order than the agent appearing in the conclusion. Our experimental results show that this cost value is higher in random networks than in graphs with a linear order, where the latter correspond to derivations such that ∀*v*_*i*_,*v*_*j*_∈*V*.(*v*_*i*_<*v*_*j*_)∨(*v*_*i*_>*v*_*j*_). In the latter ones, a higher number of transitively valid relation (due to the totality of the graph) means fewer instances of the *verify*_*high* rule are applied. For the case of distrust costs, Lemma 3 states that, given a fixed number of lazy agents, the value of distrust instances is only due to the applications of the *unverified_contra* rule. The explanation above, mapping order relation to topologies, holds in this latter case as well.

### Rankings

In this section we offer an analysis of results based on the ranking of the seeding nodes. We consider the correlation between ranking and consensus in scale-free networks and investigate:

1. whether a strictly higher ranking for one of the seeding nodes implies a greater chance to obtain a unanimous graph labelled by the same formula;
2. which type of scale-free networks (between overly sceptic, balanced and overly lazy) has the higher probability to reach a unanimous graph from a seed with higher ranking.

**Fig. 15:**
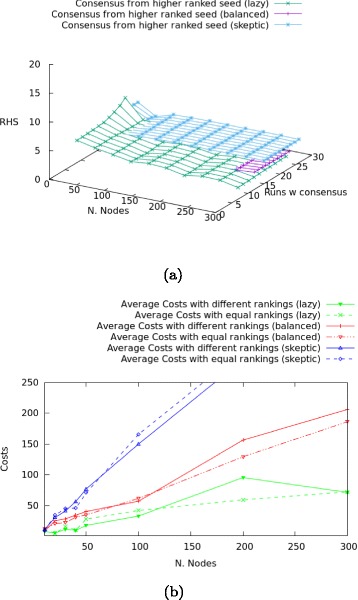
**a** Higher ranked seeds in consensus reaching network. **b** costs and ranking

The results of our analysis are plotted in Fig. 15 a. This plot reports the proportion of runs that reach consensus about a label from a higher ranked seed (the RHS axis). Results can be summarised as follows:

- There is no strict correlation between a highly ranked seed and the labelling of the network: the number of cases where the consensus is reached *and* the label is the same as the one from the higher ranked seed, is relatively small (min $\frac {1}{7}$, max $\frac {8}{26}$).
- An overly sceptic scale-free network offers the highest probability to obtain a unanimous graph labelled with the input of the higher ranked node among the seeds; the comparison between the lazy and the balanced network sees the former obtain better results in general, and the latter only for significantly large networks.

The next data analysis concerns the correlation of ranking of the seeds with costs: is information transmission from equally ranked seeds more or less expensive than transmission from differently ranked ones? The results for overly lazy, balanced and overly sceptic scale-free networks are plotted in Fig. 15 b. The results can be summarised as follows:

- Contradictory information transmission from differently ranked nodes tends to be more expensive than from equally ranked nodes in balanced and overly lazy networks: here the costs are induced by a less stable labelling for the information transmitted by higher ranked nodes.
- In lazy networks, the higher costs of differently ranked seeds tend to collapse for maximally large networks, where the costs are less than the corresponding seeding with equally ranked nodes.
- Contradictory information transmission from equally ranked nodes tends to be more expensive than from differently ranked nodes in overly sceptic networks: this can be a symptom of the greater overall epistemic balance of the sources spreading information, combined with the more common attitude of agents to require confirmation.

### Distrust and epistemic attitude

**Fig. 16:**
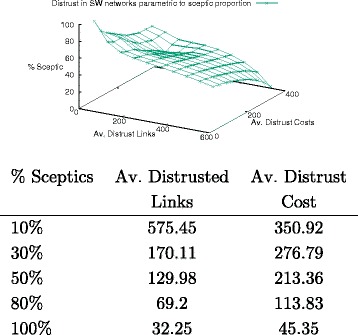
Distrust behaviour and epistemic attitude

In this and the following experiments, we focus on scale-free networks only and their distrust behaviour. First, we consider distrust as a parameter of the proportion of lazy agents in a network of 300 nodes, with a random assignment of labels to seeding agents (lazy/sceptic). As shown in Fig. 16, there is a strict correlation between the proportion of sceptic and the distrust behaviour: the more lazy agents are present in the network, the higher its overall distrust value. While this is obvious in view of the algorithm design, it is interesting to remark that in the case of a fully sceptic network (where no lazy agents are allowed), the value of distrust is to be associated entirely with the presence of contradictory information, and hence it can be used as a parameter of contradiction diffusion. The associated Table offers average values over 100 runs. It illustrates that conflict resolution is responsible on average for roughly 10% of the network’s distrusted edges, with costs averaging at around $\frac {1}{7}$ of those of a highly lazy network (i.e., with 10% of sceptic agents).

**Fig. 17:**
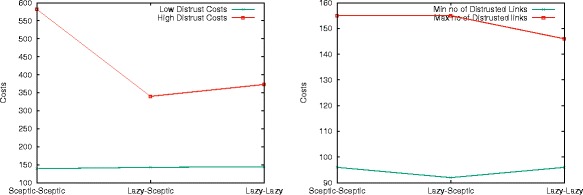
Initial nodes’ epistemic attitudes and distrust

We now extract the values for a balanced network (i.e., with 50% of sceptic agents) and compare them to the initial distribution of seeds qualified as lazy-sceptic agents. As Fig. 17 shows, there is a strict correlation of the final distribution of distrust values with the initial condition of the network: the range of minimal values for both distrust costs and number of distrusted links is relatively stable, while their maximum value decreases when moving from a configuration that has two sceptic agents as initial nodes to one that has two lazy ones. The result on distrust across the network is less influenced by the role of agents *distributing* the information than by the role of agents *receiving* it.

### Time complexity

The final analysis concerns the time complexity of our algorithm. Its running time efficiency is computed as a function relating the size of the network (in terms of the number of nodes) with the number of steps required for termination. Recall that each step in the simulation expresses one message transmission or epistemic operation of verification or distrust. We wish to know whether and in which way the network topology affects this relation.

**Fig. 18:**
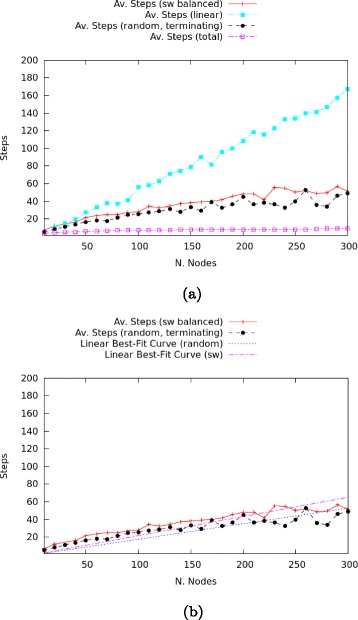
**a** Time Complexity. **b** Best fit comparisons
- Scale-free are at most as expensive as random ones.
- Total networks have a linear increase of the computational complexity in the number of nodes and require the shortest time to terminate.

Obviously, time complexity of an overly lazy scale-free network will be lower than that of a balanced one, as a result of the lower number of confirmation steps that are required to fully label the graph. Correspondingly, time complexity of an overly sceptic network will be higher, as a result of the higher number of confirmation steps required to fully label the graph. Instead, we concentrate on the values of a balanced scale-free network and compare them against linear, random and total networks. We include additional data points (every 10 nodes between 10 and 300) in order to increase precision, see Fig. 18 a. For the random network, in view of the time-out conditions mentioned in “Consensus” section, the graph only reports the values for the terminating runs. The results can be summarised as follows:

- Linear networks are the most computationally expensive in terms of time it takes for the procedure to terminate.

The difference between total networks (the cheapest one) and linear networks(the most expensive one) is over 150 steps. The algorithm has complexity *O*(*n*), see Fig. 18 b for best fit of a linear function to the data for scale-free and random networks.

## Conclusions

We have offered an agent-based modelling of contradictory information transmission across a network. Agents are heterogeneously qualified as either sceptic or lazy, and they are ranked. This model simulates some typical real case scenarios, like those of social networks or (role-based) access control systems. We consider in particular networks with memory, where the result of a given transmission in terms of trusted edges is preserved at the next transmission and the new labelling can therefore be compared. Our algorithm associates costs to confirmation processes. We identify trust as a property of communications (rather than as a relation), when such confirmation is performed. We focus on contradiction resolution by a trustworthiness metric, computed by the popularity of the information in the reachable network and the ranking of the associated node. We further compare this resolution strategy with a another metric based on distrust, where the least trusted content is rejected.

Our results suggest that a sceptic approach is favourable when maximisation of consensus is the goal; a lazy approach should be pursued when minimisation of costs is the goal. We have also suggested that ranking of initial nodes is only of little relevance to consensus reaching, while a rigidly structured network (linear) is the most expensive in this respect. Finally, in the comparison between trust and distrust, it clearly results that the former is a better mean to information propagation than the latter. Moreover, we have highlighted how the presence of contradictory information is by itself the cause of distrust generation, independently from the initial attitude of the agents.

While the simulated model allows for change of such attitude, by determining confirmation and rejection rates of sceptic and lazy agents, in future extensions, we plan to allow agents to reject information explicitly and in turn allow re-labelling. This could imply a one-time refutation on the transmission channel or a permanent one in networks with memory. The effects of such operations on the various types of networks is unknown and would offer an important opening in the analysis of negative concepts for computational trust in multi-agent systems. The dynamic of agents can be further extended by allowing change of their epistemic status (sceptic vs. lazy) after a sufficient number of (un)successful interactions (i.e., not by some pre-fixed rate).

The present analysis also misses a finer-grained analysis of structural conditions under which certain results (e.g. higher epistemic costs and consensus) are obtained. A more systematic analysis would allow to prune isomorphic networks (e.g. in view of the initial edge structure and ranking). Currently, applications of an extension of this model are being explored in the context of swarm robotics, see for example (Paudel and Clark 2016).
